# The Incorporated Society of Trained Masseuses

**Published:** 1916-02-19

**Authors:** 


### THE STATUS AND TEACHING OF MASSAGE.

### The Incorporated Society of Trained Masseuses.

The history of the Incorporated Society of Trained Masseuses should be of special interest just now, because it has done for massage workers what the proposed College of Nursing hopes to do on a larger scale for nurses. That the Society has succeeded beyond the dreams of its founders must greatly encourage all those who are trying to grapple with similar problems in the organisation of the nursing profession. Twenty-two years ago, when the Incorporated Society of Trained Masseuses was founded, the outlook in the massage world was a very serious one, for, while there was a steady demand for capable masseuses, and there were also a number of women well suited for the work, the opportunities for training were few and inadequate. There were, it is true, a few schools and teachers who were able to give a careful practical training, but instruction in the theory of treatment, anatomy, and physiology was very difficult to obtain.

The books on the subject were few, and conscientious workers felt they could work more efficiently if a more thorough training in these subjects was obtainable. But that was not all. Massage schools existed which undertook to give a certificate after a few easy lessons, and it was not uncommon for a woman to display a brass door-plate bearing the legend "Certified Masseuse" on the strength of such a certificate. The natural consequence was that such a quick and easy way of earning a living attracted quite a number of ignorant and undesirable persons to this work, and the genuine masseuse found that she was often confused in the mind of the public with a type of woman quite ignorant of any standards of professional conduct, if not with quacks and humbugs. This state of things was in the highest degree detrimental, both to the patient, who needed skilled massage, and to the genuine masseuse, who saw her occupation falling into disrepute.

It was under these circumstances that a small group of trained nurses and masseuses decided that they must organise themselves to raise the standard of massage work, and to make it a safe and desirable occupation for educated women. Having obtained the support of a large number of well-known medical men, they formed the Incorporated Society of Trained Masseuses. Provision was made for training, for the examination of candidates, for the granting of certificates, the supply of lectures and demonstrations, for a reference library, for keeping a roll of members, and the maintenance of a centre to which members could apply for advice and help. The principal rules were as follows:—

1. Not to undertake any cases of massage except under the direction of a registered medical practitioner, and in regard to massage for men to act in accordance with the by-laws of the Society.

2. Not to advertise in any way whatever, except in recognised medical papers.

3. Not to sell goods to patients in a professional capacity.

All candidates must sign these rules, and an undertaking to surrender their certificate, if in the opinion of the Council they shall be deemed guilty of conduct, professional or otherwise, detrimental to the Society. In the history of the Society, very few such cases have occurred.

It was a very small beginning; at the first examination only five candidates presented themselves, and at first the work did not increase very rapidly, the motto of the Society, "Digna sequens," was not compatible with rapid growth and instant success. The standard of anatomy and physiology required, the ruthlessness with which all but the absolutely well-grounded candidates were plucked, made slow going; however, the ever-present temptation to lower the standard of membership was steadfastly resisted, and every year saw the Society a rung higher on the ladder of public confidence.

It was in 1905 that the Army medical authorities requested the Society to hold an examination for nursing orderlies who had been taught massage by an Army sister who was a member of the Society, and from that date examinations have been held twice a year, either at Netley or Millbank, till the war made it necessary to discontinue them—and it is interesting to know that some of the blind soldiers from St. Dunstan's Hospital are now preparing for the Society's examination. Another landmark was passed in 1909, when, the number of candidates in London having risen to between 200 and 300, the examination began to be held in the Examination Hall of the Royal Colleges of Physicians and Surgeons, while an examination was also held in Dublin, which continues twice yearly to the present time. The same year saw a Swedish Remedial Exercises Examination instituted in London, which was followed a few years later by an examination for a Diploma of Teacher of Medical Gymnastics and Massage, while last year Medical Electricity was added to the list of examinations organised by the Society. Candidates are only allowed to enter for these examinations if trained in schools, now over 100 in number, inspected and approved by the Society. The total number of certificate holders is now over 3,300.

The holding of examinations is, however, only one of the functions of this Society. The improvement of opportunities for study has claimed a large share of the attention of the Council; through its efforts the London School of Medicine for Women has been induced to allow massage students to attend its anatomy classes, and at the present time seventy candidates and members are attending there. The use of Guy's Hospital Museum is also allowed them at stated times, while lectures and demonstrations are constantly being given in the

Society's large and comfortable club-room, which adjoins its offices in Great Portland Street, where also is to be found a good reference library, which is of incalculable value alike to candidates for examination and practising masseuses, while the monthly Journal plays a very important part in keeping country members in touch with new developments and modern methods of treatment.

The Council of the Incorporated Society is elected by the members and consists of six massage teachers, six practising masseuses, and some three or four co-opted members. No appeal has ever been made for funds; the Society has always paid its way.

In matters of public welfare the Society has also played a part, as in the case of the General Powers Bill, when the Chairman of Council (Miss Robinson) gave evidence before a Select Committee of both Houses of Parliament in support of the petition of the London County Council for the enlargement of its powers to include the registration and inspection of massage establishments. As a result the inspection of these establishments falls under the control of the L.C.C. instead of that of the twenty-nine Borough Councils, as originally proposed. That such a comparatively small and uninfluential body of women has been able to organise itself so successfully lends support to the hope that a similar organisation for nurses, if built on sound lines and administered with the same dogged resolve to uphold the best traditions of its members, should be able to reduce to order the state of chaos which to-day exists in the nursing world, and to constitute a body which, like the Incorporated Society of Trained Masseuses, is capable of voicing the opinion of its members in public matters which vitally affect their welfare or that of the sick whom they serve.

